# Synergistic effects of S100 calcium-binding protein A12 combined with Pentraxin 3 in invasive pulmonary aspergillosis and their clinical application prospects

**DOI:** 10.3389/fcimb.2026.1767035

**Published:** 2026-03-05

**Authors:** Xiao-Lian Zhou, Xiao-Bo Hu, Hui Liu

**Affiliations:** 1 Department of Pulmonary and Critical Care Medicine, West China School of Medicine, Sichuan University, Sichuan University Affiliated Chengdu Second People’s Hospital, Chengdu Second People’s Hospital., Chengdu, Sichuan, China; 2 Department of Emergency Medicine, The Affiliated Hospital of Southwest Medical University, Luzhou, Sichuan, China

**Keywords:** clinical application prospects, IPA, PTX3, S100A12, synergistic effect

## Abstract

Invasive pulmonary aspergillosis (IPA) is a severe deep-seated fungal infection caused by *fungi* of the genus *Aspergillus*. In recent years, its global incidence has shown a marked upward trend, posing a serious threat especially to immunocompromised patients, such as hematopoietic stem cell transplant recipients, cancer patients undergoing chemotherapy, and individuals on long-term glucocorticoid therapy. The core clinical dilemmas lie in the difficulty of early diagnosis and the narrow therapeutic window. Currently used clinical diagnostic indicators, including the galactomannan (GM) assay, (1,3)-β-D-glucan(G) assay, and imaging examinations, suffer from insufficient sensitivity or specificity, while traditional microbiological detection methods have a relatively long turnaround time. S100 calcium-binding protein A12 (S100A12) and Pentraxin 3 (PTX3) are both key molecules in the innate immune response of the human body, playing central roles in the immune regulation of infectious diseases. Recent studies have demonstrated that both molecules are abnormally expressed in IPA patients and may participate in the processes of *Aspergillus* infection recognition, immune clearance, and inflammatory regulation through synergistic effects, thereby providing new directions for the early diagnosis, disease assessment, and targeted therapy of IPA. This review will systematically elaborate on the molecular characteristics of S100A12 and PTX3, explore their synergistic mechanism and combined diagnostic value in IPA, and analyze their prospects for clinical application.

## 1 Introduction

**Figure 1:**
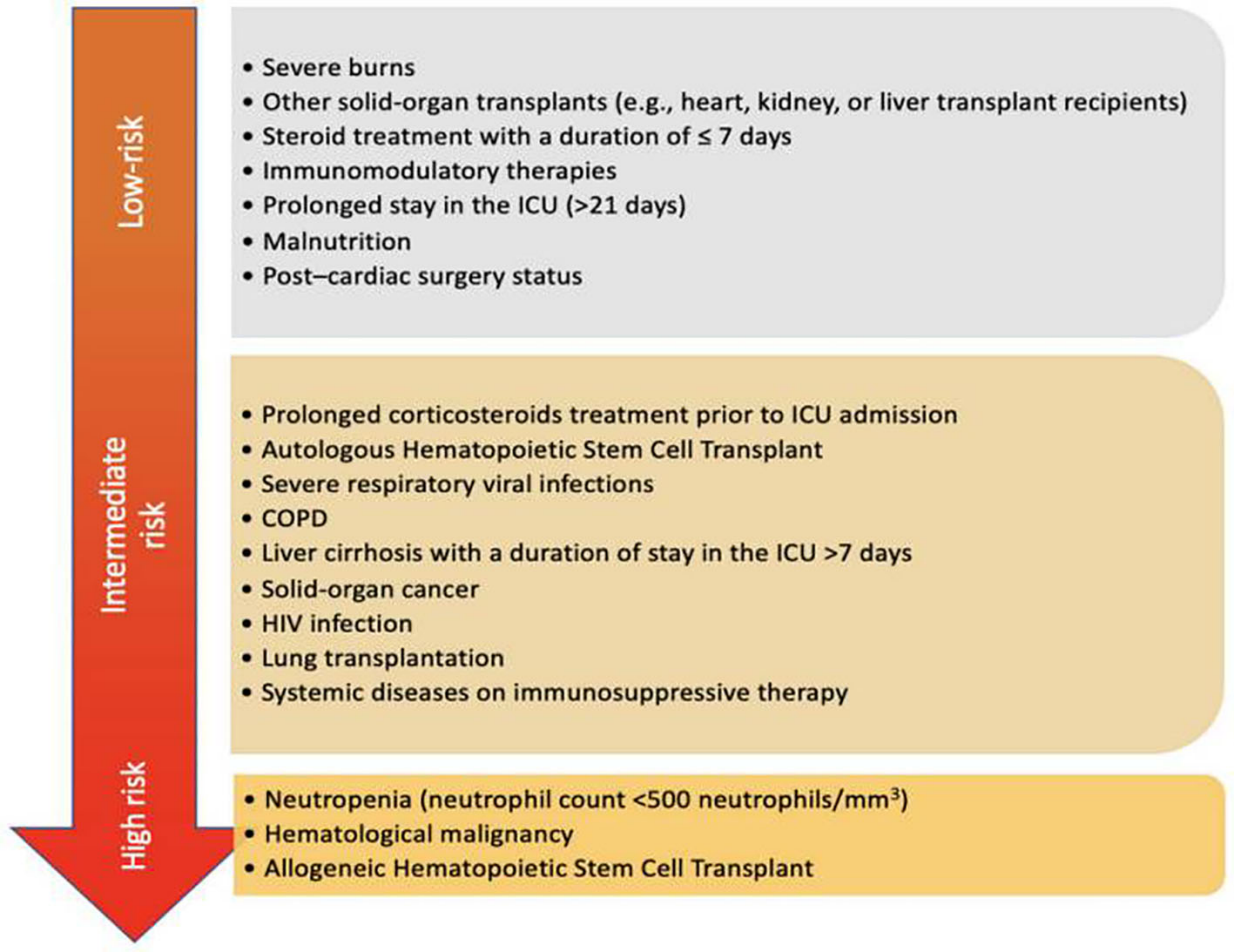
Risk factors for IPA in the ICU.

*Aspergillus* species are opportunistic pathogens, with the main pathogenic populations including *Aspergillus fumigatus*, *Aspergillus flavus*, and *Aspergillus niger*. An epidemiological survey showed that the distribution of *Aspergillus* in the local region is dominated by *Aspergillus fumigatus* (56.41%), followed by *Aspergillus flavus* (20.51%) and *Aspergillus niger* (15.38%) as the secondary pathogenic species (Yuening et al., 2020). In recent years, the detection rate of cryptic *Aspergillus* species (such as *Aspergillus thermomutatus* and *Aspergillus terreus*) has been gradually increasing among transplant recipients (Kimura and Husain, 2025). Infection is mainly caused by inhalation of environmental *Aspergillus* spores through the respiratory tract, while pulmonary infection via hematogenous spread or direct extension is extremely rare. Based on infection patterns and imaging features, IPA is mainly classified into four types: angioinvasive type, airway-invasive type, chronic necrotizing type, and aspergilloma (mostly secondary to pulmonary cavitary lesions) (Expert consensus on the diagnosis and treatment of pulmonary fungal diseases, 2007). Invasive aspergillosis is a major cause of morbidity and mortality in patients with neutropenia, hematological malignancies, and organ transplants. It has an insidious onset, rapid progression, and high fatality rate. Denning DW reported in a study that the crude mortality rate of invasive aspergillosis (with IPA as the main type) in ICU patients is approximately 46%–82%, and the attributable mortality rate is about 51% (Denning, 2024). A retrospective database study excluded patients with cancer, transplantation, neutropenia, and immunodeficiency, enrolled 412 aspergillosis patients, and reported that the in-hospital mortality rate decreased to 46% (Baddley et al., 2013). This indicates that the combination of severe illness, comorbid underlying diseases, and invasive aspergillosis may contribute to the highest mortality rate. Elkhapery et al. summarized the risk factors of IPA in the ICU (see **Figure 1**) (Elkhapery et al., 2025). The incidence of IPA is significantly higher in immunocompromised populations. The 2023 edition of the Chinese Expert Consensus on Invasive Fungal Diseases after Hematopoietic Stem Cell Transplantation indicated that the incidence of IPA is approximately 7.8%–13.1% in allogeneic hematopoietic stem cell transplant recipients, 1.2%–4.0% in autologous hematopoietic stem cell transplant recipients (Chinese expert consensus for invasive fungal disease in patients after hematopoietic stem cell transplantation (2023), 2023), and 8%–11% in hematological malignancy patients undergoing chemotherapy (Bitterman et al., 2019). IPA is rare in the general population and mostly occurs as sporadic cases. Consistent with the overall high-risk groups for invasive aspergillosis, the core high- risk groups for IPA include hematopoietic stem cell transplant recipients, solid organ transplant recipients, long -term users of glucocorticoids/immunosuppressants, and patients with hematological malignancies. Early diagnosis of IPA is challenging. Currently, in addition to conventional detection methods such as the galactomannan (GM) assay, (1,3)-β-D-glucan(G) assay, researchers are exploring novel specific detection targets, including imaging examinations, interleukin-17 (IL-17), *Aspergillus*-specific IgM antibody, S100A12, and PTX3. This review will systematically elaborate on the synergistic mechanism of S100A12 and PTX3 in IPA, providing evidence for early diagnosis, disease assessment, and prognosis judgment in high-risk populations.

## 2 Molecular functions of S100A12 and PTX3 and their core roles in infections

### 2.1 Biological characteristics and functions of S100A12 and PTX3

**Figure 2:**
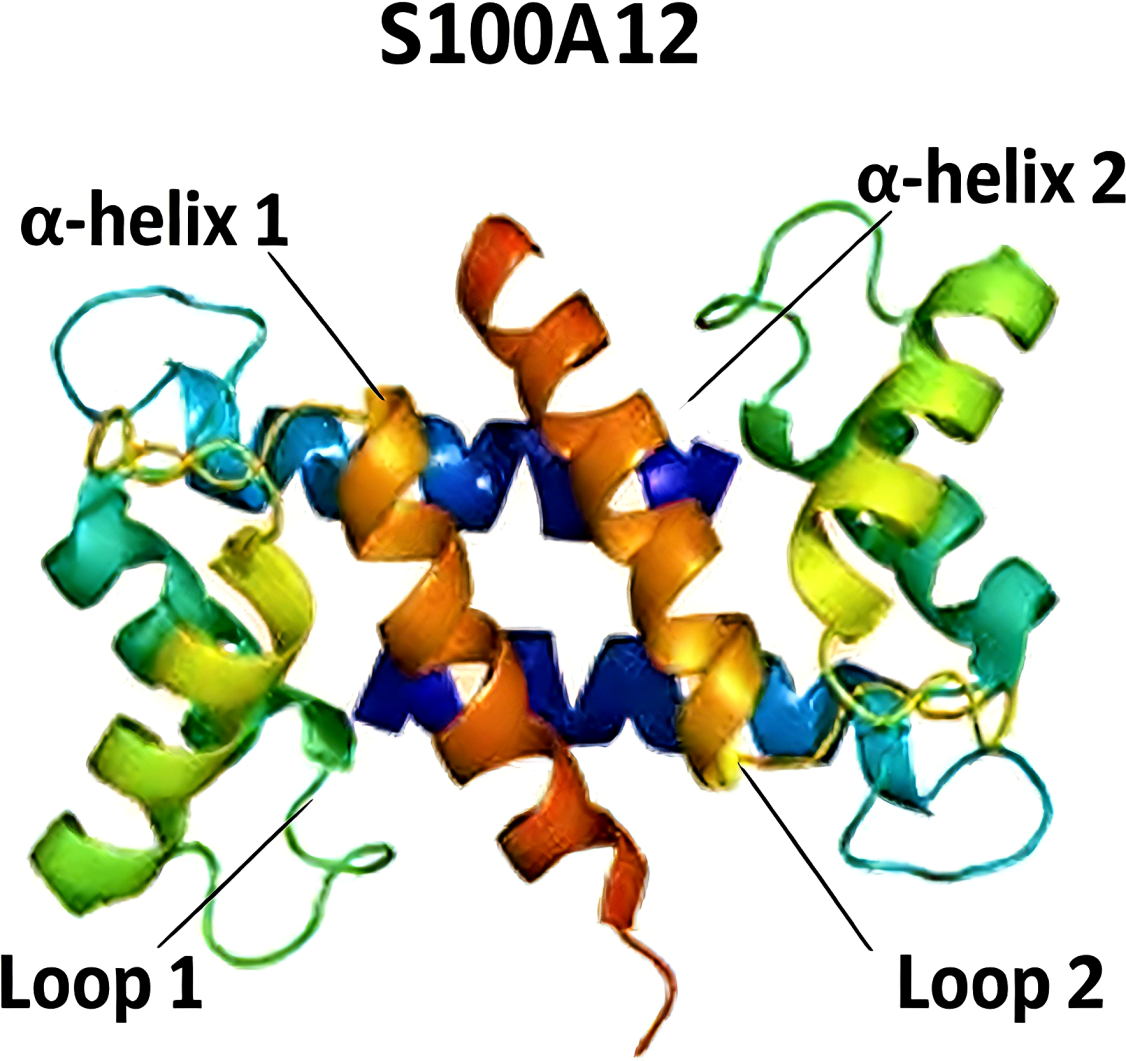
Molecular structure of S100A12.

S100A12 is a key member of the S100 calcium-binding protein family. The S100 proteins were first discovered in bovine brain by Moore in 1965, and subsequently, S100A12 was identified in pigs, dogs and cattle by Guignard in 1995 (Meijer et al., 2012). S100A12 has a relative molecular mass of 10575 Da, with its monomer consisting of 92 amino acids. The core structure of S100A12 is a homodimer, formed by the non-covalent polymerization of two subunits with an identical amino acid sequence. A single subunit contains a typical EF-hand fold structure, comprising two α-helical domains at the N-terminus and C-terminus; these two domains are linked by a flexible hinge region to form two independent EF-hand motifs (see **Figure 2**). S100A12 acts as an extracellular newly identified receptor for advanced glycation end products (en-RAGE) (Mori et al., 2009). Its molecular structure contains an EF-hand calcium-binding domain, which can undergo conformational changes upon Ca^2+^ binding and then interact with target proteins to exert biological effects. S100A12 is mainly secreted by neutrophils, monocytes and macrophages.

**Figure 3:**
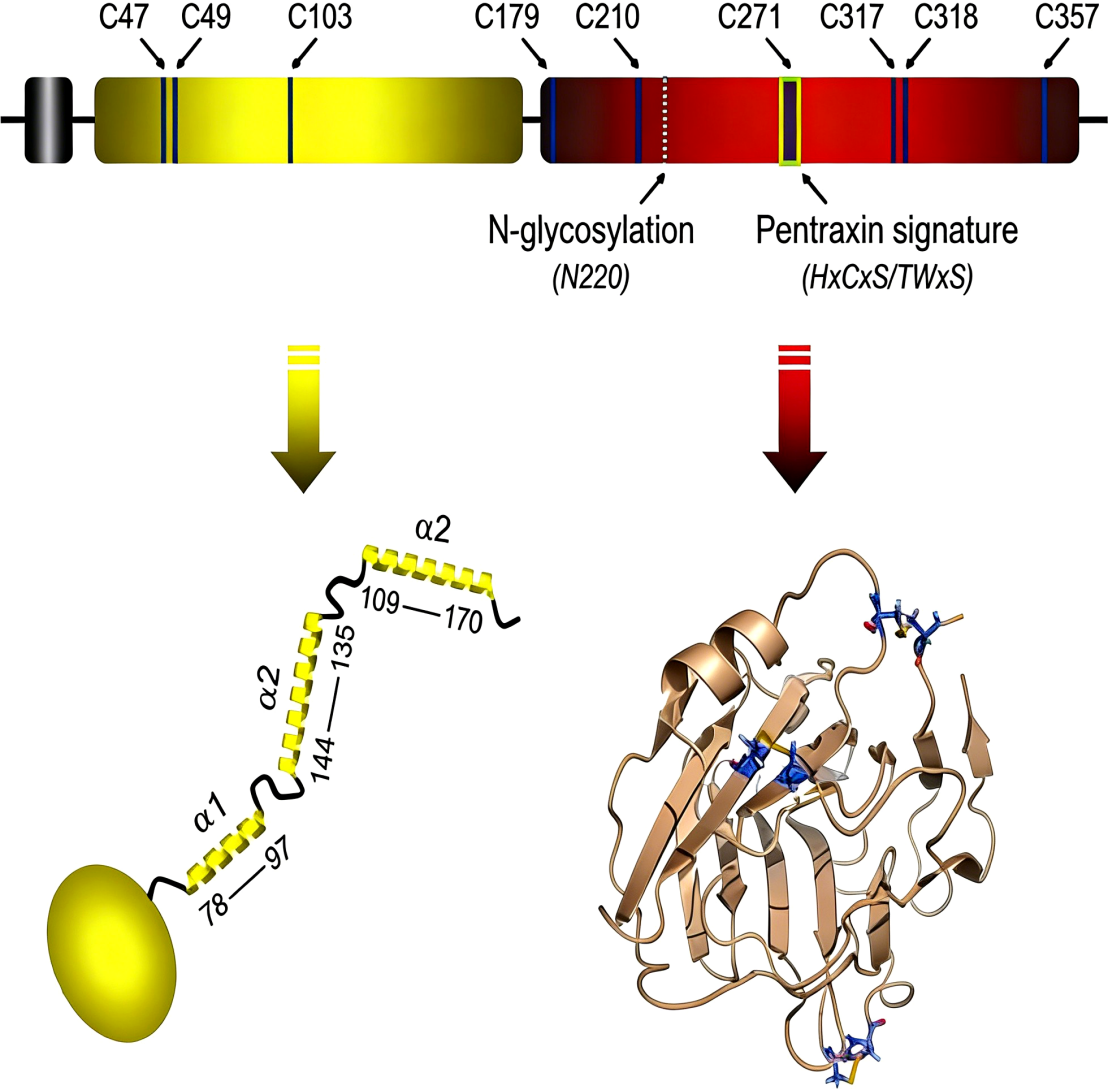
Molecular structure of PTX3.

Pentraxins represent an evolutionarily conserved protein superfamily characterized by the pentraxin domain. PTX3 is a multimeric glycoprotein with a molecular mass of 340 kDa, and its subunit is composed of 381 amino acids. PTX3 consists of a unique N-terminal region and a C-terminal domain containing 203 amino acids (see **Figure 3**) (Inforzato et al., 2012). As a representative member of the long pentraxin family, PTX3 and the classical C-reactive protein (CRP) are both pattern recognition receptors (PRRs). PTX3 is an acute-phase reactant, with relatively low basal levels in the blood under normal physiological conditions (approximately 25 ng/mL in mice and < 2 ng/mL in humans) (Zhang et al., 2024). PTX3 is inducibly produced in cells of the myelomonocytic lineage, namely monocytes, macrophages and myeloid dendritic cells, in response to the proinflammatory cytokines IL-1β and TNF-α as well as a variety of other stimuli (Daigo et al., 2016; Garlanda et al., 2018).

### 2.2 Regulatory roles of S100A12 and PTX3 in infections

S100A12 participates in immune regulation and host defense responses through multiple molecular mechanisms. As a damage-associated molecular pattern (DAMP), S100A12 exerts dual effects in infection and inflammatory responses. On the one hand, it binds to the receptor for advanced glycation end products (RAGE) to activate the NF-κB signaling pathway, thereby promoting the release of proinflammatory cytokines such as IL-6 and TNF-α, and enhancing local inflammatory responses to eliminate pathogens (Haiying and Luoyang, 2015). In terms of antifungal mechanisms, Sanhita et al. demonstrated that S100A12 has a direct membrane-disrupting effect: it can bind to phospholipid components of fungal membranes and destroy membrane integrity. Additionally, S100A12 induces reactive oxygen species (ROS) production and mitochondrial dysfunction, and affects the progression of the fungal cell cycle through its DNA-binding capacity (R oy et al., 2024). Cunden et al. reported the discovery that S100A12 exhibits *in vitro* antifungal activity, which is attributed to zinc deprivation in *fungi* (Cunden et al., 2016).

PTX3 exists in a pentameric form and exerts multiple antifungal effects by recognizing specific antigens (e.g., galactomannan) on the fungal cell wall. First, as an opsonin, PTX3 activates the classical complement pathway and forms complexes with complement proteins C1q, C3b, and surfactant protein D, thereby enhancing complement-mediated fungal lysis (Inforzato et al., 2006; Dellière et al., 2024). Second, Zhang J et al. confirmed using corneal epithelial cells infected with *Aspergillus fumigatus* that PTX3 can directly bind to *Aspergillus* spores and hyphae, inhibiting their germination and invasive ability (Zhang et al., 2018). Third, PTX3 promotes the phagocytic activity of phagocytes against *Aspergillus* by binding to Fcγ receptors on the surface of phagocytes. Studies have shown that PTX3 gene-knockout mice exhibit significantly increased pulmonary fungal burden and higher mortality after *Aspergillus* infection, confirming that PTX3 is indispensable for host defense against *Aspergillus* infection (Moalli et al., 2010; Yuening et al., 2020).

## 3 Synergistic mechanisms of S100A12 and PTX3 in invasive pulmonary aspergillosis

Although S100A12 and PTX3 belong to different molecular families, they do not function independently in the immune response against IPA. Instead, they may form a complete antifungal immune regulatory network through a synergistic “recognition-signal transduction-effect amplification” mode. The exploration of their synergistic mechanisms is mainly reflected in the following three aspects:

### 3.1 Synergistic recognition of *Aspergillus* pathogens to enhance targeted immune responses

The cell wall of *Aspergillus* has a complex composition, containing a variety of antigenic molecules such as glucan, galactomannan, and chitin (Bernard and Latgé, 2001), making it difficult for a single pattern recognition receptor (PRR) to achieve comprehensive and efficient pathogen recognition. In a study on fungal keratitis, Roy et al. found that S100A12 mainly binds to phospholipids in the fungal cell membrane, inducing membrane permeabilization and early disruption of membrane potential, thereby inhibiting the hyphal formation of *Fusarium* (R oy et al., 2024). Given that *Fusarium* and *Aspergillus* are highly similar in core aspects including cellular structure, pathogenic characteristics, host immune responses, and antifungal action targets, it is speculated that the antifungal mechanism of S100A12 against *Aspergillus* is analogous to that against *Fusarium*. In contrast, PTX3 specifically recognizes galactomannan (GM) and galactosamine-galactan (GAG) in the fungal cell wall (Dellière et al., 2024). The two proteins form a synergistic recognition mode by targeting different cellular components of *Aspergillus*, which significantly improves the efficiency of host recognition of the pathogen. Multiple experiments have confirmed that both S100A12 and PTX3 can bind to *Aspergillus* conidia, further enhancing the chemotactic and phagocytic activity of phagocytes against conidia and reducing the likelihood of pathogen escape. Therefore, their synergistic recognition can also avoid immune tolerance caused by the recognition defect of a single receptor, laying a foundation for the initiation of subsequent immune effector responses.

### 3.2 Synergistic activation of immune signaling pathways to amplify inflammatory responses

**Figure 4:**
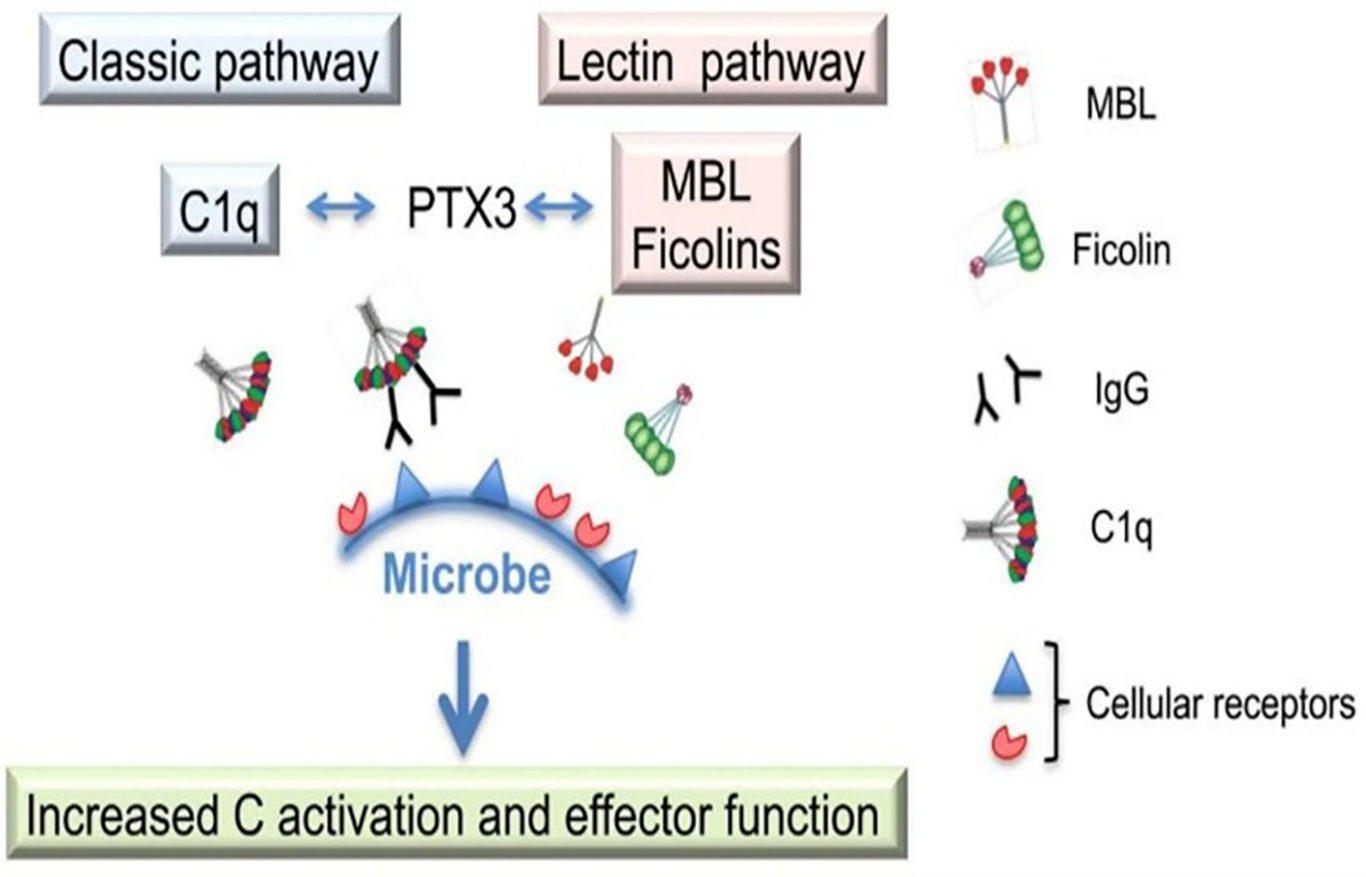
PTX3 interacts with other fluid-phase pattern recognition molecules, namely complement component 1q (C1q), mannose-binding lectin (MBL) and ficolins, via the classical and lectin pathways to enhance microbial recognition, promote complement activation and augment effector functions.

S100A12 and PTX3 can form an effector amplification loop of the inflammatory response by jointly activating the NF-κB signaling pathway. Upon binding to RAGE, S100A12 can phosphorylate the NF-κB p65 subunit via activating the downstream MAPK pathway, thereby promoting its nuclear translocation and initiating the transcription of proinflammatory factors (Ye, 2025). In contrast, after binding to pathogens, PTX3 can interact with Toll-Like Receptor 4 (TLR4) to enhance the TLR4-mediated NF-κB activation signal and further amplify the inflammatory response (Qi et al., 2020). Cecilia et al. found that PTX3 interacts with other fluid-phase pattern recognition molecules, namely complement component 1q (C1q), mannose-binding lectin (MBL), and ficolins, which enriches the capacity for microbial recognition and promotes complement activation and the enhancement of effector functions (see **Figure 4**) (Garlanda et al., 2018). In a murine study, Shankar et al. observed that PTX3 can activate the complement pathway, leading to increased expression of chemotactic complement factors such as C-type lectins and TLRs, as well as elevated expression of mannose-binding lectin. MBL can activate the complement system through the alternative pathway in response to the binding of mannose to the fungal cell wall (Shankar et al., 2018).

### 3.3 Synergistic regulation of phagocyte functions to improve pathogen clearance efficiency

Phagocytes (neutrophils and macrophages) are the core effector cells for eliminating *Aspergillus*, and S100A12 and PTX3 can synergistically enhance their functions through distinct pathways. On the one hand, S100A12 can promote the production of reactive oxygen species (ROS) in phagocytes via the RAGE signaling pathway, thereby enhancing oxidative bactericidal activity (Hofmann Bowman and Schmidt, 2011). On the other hand, S100A12 binds to fungal membrane phospholipids to inhibit the expression of fungal virulence factors, which in turn suppresses hyphal formation and reduces invasive damage to host tissues, creating favorable conditions for phagocytes to clear pathogens (R oy et al., 2024). Liu et al. demonstrated that the transcription factor CCAAT/enhancer-binding protein δ (CEBPD) is upregulated during *Aspergillus fumigatus* conidial infection, and this factor can activate PTX3 expression by directly binding to the promoter region of the *PTX3* gene, thereby enhancing the phagocytic capacity of macrophages against *Aspergillus fumigatus* conidia. This finding clarifies the critical role of PTX3 in the antifungal phagocytic activity of macrophages (Liu et al., 2021). Multiple studies have indicated that both S100A12 and PTX3 induce the release of proinflammatory factors by regulating the inflammatory microenvironment during infection, and the inflammatory pathways they modulate overlap. Such pathway synergy suggests that the two proteins are highly likely to synergistically potentiate phagocyte functions and improve pathogen clearance efficiency in antifungal immunity.

## 4 Clinical application prospects of S100A12 combined with PTX3 in IPA

Based on the synergistic mechanism of S100A12 and PTX3 in IPA, the combined detection and targeted regulation of these two molecules show broad prospects in the clinical management of IPA, which are specifically reflected in four aspects: early diagnosis, disease assessment, prognosis evaluation, and targeted therapy.

### 4.1 As combined diagnostic biomarkers to improve the efficacy of early diagnosis of IPA

Early diagnosis of invasive pulmonary aspergillosis (IPA) is the key to reducing mortality, yet conventional diagnostic methods have notable limitations: *Aspergillus* culture has a long turnaround time (3–7 days), and its diagnostic sensitivity is highly influenced by specimen type, with low positive rates observed in samples such as blood and bronchoalveolar lavage fluid (BALF); the galactomannan (GM) assay has a relatively high false-positive rate—serum GM testing yields a false-positive rate of 66.7% in patients receiving cefoperazone/sulbactam, and false positives are also common with β-lactam antibiotics (Niu et al., 2020); chest computed tomography (CT) lacks specific findings in the early stage of infection. A series of *in vitro* experiments have demonstrated that S100A12 activates human monocytes to promote inflammatory responses upon binding to specific receptors, and serum S100A12 levels increase within hours to 1–2 days post-infection (Foell et al., 2013). Zhang et al. found in a rat corneal model that PTX3 mRNA expression begins to rise at 8 hours, peaks at 16 hours, and declines at 24 hours following *Aspergillus fumigatus* infection (Zhang et al., 2018). A clinical study of patients with aspergillosis showed that receiver operating characteristic (ROC) curve analysis revealed plasma PTX3 had a sensitivity of 79.3% and a specificity of 72.1% for the diagnosis of pulmonary aspergillosis (Li, 2018). Qian H et al. also confirmed in a clinical study that PTX3 levels in BALF have superior diagnostic sensitivity and specificity compared with the conventional GM assay, particularly in patients with severe immunocompromise, providing important clues for the early diagnosis of IPA (Qian et al., 2021). Kałużna et al. found in a clinical study that serum concentrations of both PTX3 and S100A12 were significantly higher in patients with Crohn’s disease than in healthy controls, and the changes in these two markers were positively correlated with disease activity in Crohn’s disease. Combined detection of the two markers can improve the specificity of Crohn’s disease diagnosis and the accuracy of disease activity assessment (Kałużna et al., 2023). Given the synergistic effect of combined PTX3 and S100A12 detection in patients with Crohn’s disease, and their similar mechanisms of action in fungal infections, further research is warranted to investigate the diagnostic efficacy of their combination in patients with IPA.

### 4.2 Application in disease severity assessment and treatment response monitoring

The expression levels of S100A12 and PTX3 are closely correlated with disease severity and treatment response in IPA patients, and thus can serve as indicators for dynamic monitoring. Zhou et al. demonstrated in a study that serum levels of PTX3 and IL-23 were significantly elevated in patients with pulmonary aspergillosis compared with healthy controls (P<0.001). The diagnostic sensitivity and specificity of serum PTX3 for pulmonary aspergillosis (PA) were 0.925 and 0.914, respectively. Cox regression analysis identified PTX3 and IL-23 as independent risk factors affecting patient prognosis (Zhou et al., 2025). During antifungal therapy, serum PTX3 levels decreased significantly in patients with a favorable treatment response, falling from 299.0 [119.5–477.0] to 138.3 [80.9–386.1] pg/mL (p < 0.05); in contrast, PTX3 levels increased in patients with adverse treatment outcomes, rising from 177.1 [117.8–318.8] to 243.0 [169.5–488.4] pg/mL (p < 0.05). Serial measurement of PTX3 may act as a useful biomarker for monitoring treatment response in invasive pulmonary aspergillosis, facilitating the identification of disease progression or recurrence (Huang et al., 2026). Sruthi et al. detected significantly upregulated expression of IL-22 and S100A12 in corneal scraping samples from patients with *Aspergillus flavus* keratitis (Mallela et al., 2021). Roy et al. showed in a murine model of corneal fungal infection that both mRNA and protein expression levels of S100A12 were markedly increased in mice with *Fusarium solani* keratitis. Given the significant elevation of S100A12 in corneal tissues infected with *F. solani*, further assays confirmed that S100A12 effectively inhibited *Fusarium solani* corneal infection in mice: fungal growth was reduced by more than 95% at a concentration of 5 μM, and by over 99% at concentrations of 10 μM, 25 μM and higher. S100A12 could significantly suppress the growth and viability of *F. solani* and a small number of other species of the *Fusarium* genus (R oy et al., 2024). It can therefore be inferred that serial measurement of S100A12 may serve as a useful biomarker for monitoring treatment response in pulmonary aspergillosis. Since PTX3 and S100A12 exhibit consistent trends in expression levels during the antifungal response, the combined dynamic monitoring of their levels enables timely evaluation of therapeutic efficacy, providing evidence for the adjustment of treatment regimens.

### 4.3 As prognostic assessment indicators to guide individualized therapy

The baseline levels and dynamic changes of S100A12 and PTX3 can effectively predict the prognosis of patients with invasive pulmonary aspergillosis (IPA). Sun et al. found that the mortality rate of IPA patients was significantly higher than that of non-IPA patients, with the 30-day mortality rate being 26.15% versus 8.38% (P<0.001) and the 90-day mortality rate being 34.36% versus 13.24% (P<0.001). Elevated levels of PTX3 in plasma and bronchoalveolar lavage fluid (BALF) were identified as an independent predictor of poor outcomes in IPA. The predictive accuracy of plasma PTX3 levels was superior to that of BALF, indicating that PTX3 is an independent predictor of 90-day mortality in IPA patients (Sun et al., 2025). In addition, the mRNA and protein expression levels of S100A12 were also significantly upregulated in patients with fungal keratitis as mentioned above. A large number of previous studies have focused on the correlation analysis between single nucleotide polymorphisms (SNPs) and the susceptibility, drug efficacy and prognosis of pulmonary aspergillosis. Jansen et al. introduced therapeutic drug monitoring with model-informed precision dosing (MIPD) for posaconazole into clinical practice in a study, which provided personalized antifungal therapy for patients at risk of invasive fungal disease by optimizing the individual posaconazole exposure (Jansen et al., 2024). Jin et al. conducted a clinical study on lung cancer patients without IPA who underwent surgical resection, and found that the interactions between Dectin-1 gene rs3901533, rs7309123 and low body weight were associated with an increased risk of IPA in lung cancer patients after surgery (Jin et al., 2025). Based on the above findings, a prognostic prediction model incorporating S100A12, PTX3 and clinical indicators (e.g., body weight, underlying diseases, drug efficacy) can be constructed, which provides evidence for the stratified management of IPA patients.

### 4.4 As targets for targeted therapy to develop novel antifungal strategies

**Figure 5:**
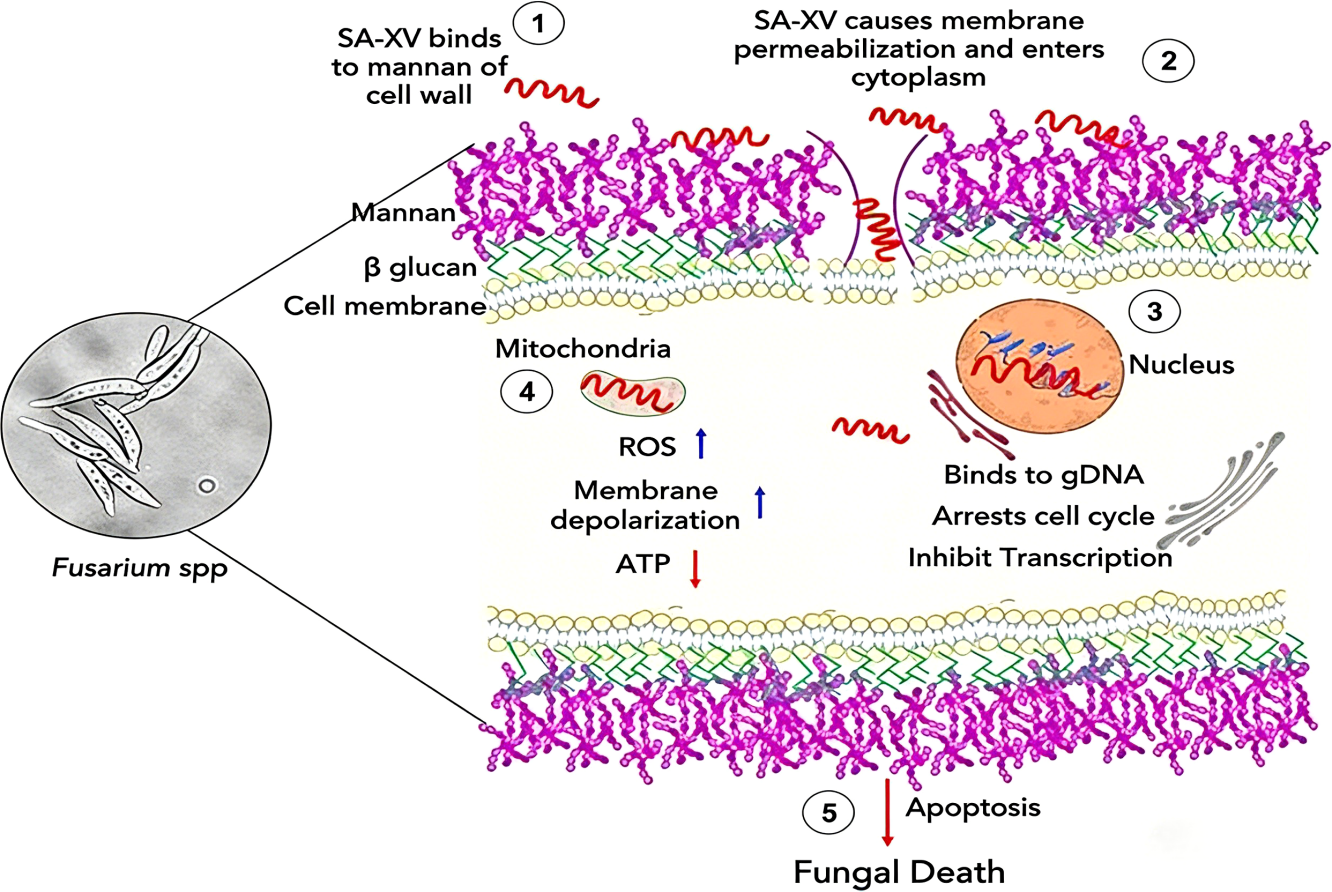
Mechanism of action of SA-XV on fungal cells. SA-XV interacts with the fungal cell wall and plasma membrane, translocates across the cell membrane and accumulates in the cytoplasm, then colocalizes to the nucleus, binds to genomic DNA (gDNA), arrests the cell cycle, targets and permeabilizes mitochondria, and ultimately induces fungal cell death via apoptosis.

Beyond serving as diagnostic and evaluative biomarkers, the mechanisms of action of S100A12 and PTX3 also offer novel directions for the targeted therapy of invasive pulmonary aspergillosis (IPA). Agarwal et al. demonstrated that SA-XV, a 15-amino-acid fragment of S100A12, can bind to phospholipids of *Fusarium* and *Candida* species. SA-XV exerts significant antifungal activity similar to that of the parent peptide against a variety of fungi, and its mechanism of action is shown in **Figure 5**. The study also found that SA-XV possesses wound-healing-promoting properties. Therefore, S100A12 represents a unique drug candidate with potential for the treatment of fungal infections, and further research will facilitate the optimization and development of S100A12 as a potential alternative novel therapy (Agarwal et al., 2025). Pietro et al. conducted a study in a rat model and showed that the combined administration of PTX3 and voriconazole enhanced synergistic antifungal activity in immunosuppressed rats induced by cortisone acetate and infected with *Aspergillus fumigatus* conidia. Monotherapy with a high dose of 1.5 mg/kg PTX3 significantly reduced the mortality rate, with a median survival time of 13 days and an interquartile range (IQR) of 8 to >21 days (P<0.05). These data confirmed that PTX3 can enhance the phagocytic capacity of innate immune cells against conidia (Lo Giudice et al., 2012). Experiments have indicated that for IPA patients with severe immunosuppression, exogenous supplementation of PTX3 and S100A12 can enhance the host’s innate immune response, thereby boosting antifungal activity (Garlanda et al., 2002). On the other hand, the common signaling pathways of the two (e.g., the NF-κB pathway) can be regulated to enhance antifungal immunity while avoiding inflammatory damage. For example, selective inhibition of S100A12-mediated excessive inflammatory responses—predominantly driven by RAGE-mediated NF-κB/MAPK activation—can be achieved without impairing its opsonophagocytic function. PTX3 regulates excessive complement activation by binding to C1q, restricts the overactivation of NF-κB, modulates the intensity of inflammation appropriately, and reduces lung tissue damage.

## 5 Challenges and prospects

Although the synergistic effects and clinical value of S100A12 and PTX3 in invasive pulmonary aspergillosis (IPA) have been initially confirmed, numerous challenges remain. First, the expression of these two molecules is not specific to IPA but may also be elevated in other pulmonary infections such as bacterial pneumonia and pulmonary tuberculosis; further exploration is needed to improve their specificity by combining them with other biomarkers (e.g., *Aspergillus*-specific IgM antibodies and the galactomannan (GM) assay). Second, the sample sizes of existing relevant studies are relatively small, and there is a particular lack of multicenter, prospective clinical research data, leading to the absence of unified reference thresholds and clinical application criteria for these biomarkers. Third, the administration routes, dosages and safety of exogenously supplemented recombinant proteins need to be further verified through clinical trials, and the development of signal pathway-targeted drugs is also confronted with issues such as low drug delivery efficiency and off-target effects.

With the development of molecular biology technologies in the future, single-cell sequencing, proteomics and other techniques can be used to thoroughly elucidate the regulatory networks of S100A12 and PTX3 in the IPA immune response and clarify their interactions with other immune molecules. Large-scale multicenter clinical studies should be conducted to establish reference ranges of these biomarkers and clinical application guidelines for different populations. In addition, nanotechnology can be integrated to develop targeted delivery systems, thereby enhancing the pulmonary targeting of recombinant proteins and signal pathway inhibitors and reducing systemic adverse reactions. Despite the many unresolved issues at present, with the deepening of research, S100A12 and PTX3 are expected to become multifunctional biomarkers for the clinical management of IPA as well as novel therapeutic targets, bringing new hope for improving the prognosis of IPA patients.
